# The Binding Sites of miR-619-5p in the mRNAs of Human and Orthologous Genes

**DOI:** 10.1186/s12864-017-3811-6

**Published:** 2017-06-01

**Authors:** Shara Atambayeva, Raigul Niyazova, Anatoliy Ivashchenko, Anna Pyrkova, Ilya Pinsky, Aigul Akimniyazova, Siegfried Labeit

**Affiliations:** 1 SRI Of Biology and Biotechnology Problems, Al-Farabi Kazakh National University, Almaty, Kazakhstan; 2 Institute for Anaesthesiology and Intensive Operative Care Medical Faculty Mannheim, Mannheim, Germany

**Keywords:** miR-619-5p, miRNA, mRNA, Gene, Human, Orthologous genes

## Abstract

**Background:**

Normally, one miRNA interacts with the mRNA of one gene. However, there are miRNAs that can bind to many mRNAs, and one mRNA can be the target of many miRNAs. This significantly complicates the study of the properties of miRNAs and their diagnostic and medical applications.

**Results:**

The search of 2,750 human microRNAs (miRNAs) binding sites in 12,175 mRNAs of human genes using the MirTarget program has been completed. For the binding sites of the miR-619-5p the hybridization free energy of the bonds was equal to 100% of the maximum potential free energy. The mRNAs of 201 human genes have complete complementary binding sites of miR-619-5p in the 3’UTR (214 sites), CDS (3 sites), and 5’UTR (4 sites). The mRNAs of *CATAD1, ICA1L*, *GK5*, *POLH,* and *PRR11* genes have six miR-619-5p binding sites, and the mRNAs of *OPA3* and *CYP20A1* genes have eight and ten binding sites, respectively. All of these miR-619-5p binding sites are located in the 3’UTRs. The miR-619-5p binding site in the 5’UTR of mRNA of human *USP29* gene is found in the mRNAs of orthologous genes of primates. Binding sites of miR-619-5p in the coding regions of mRNAs of *C8H8orf44, C8orf44,* and *ISY1* genes encode the WLMPVIP oligopeptide, which is present in the orthologous proteins. Binding sites of miR-619-5p in the mRNAs of transcription factor genes *ZNF429* and *ZNF429* encode the AHACNP oligopeptide in another reading frame. Binding sites of miR-619-5p in the 3’UTRs of all human target genes are also present in the 3’UTRs of orthologous genes of mammals. The completely complementary binding sites for miR-619-5p are conservative in the orthologous mammalian genes.

**Conclusions:**

The majority of miR-619-5p binding sites are located in the 3’UTRs but some genes have miRNA binding sites in the 5’UTRs of mRNAs. Several genes have binding sites for miRNAs in the CDSs that are read in different open reading frames. Identical nucleotide sequences of binding sites encode different amino acids in different proteins. The binding sites of miR-619-5p in 3’UTRs, 5’UTRs and CDSs are conservative in the orthologous mammalian genes.

**Electronic supplementary material:**

The online version of this article (doi:10.1186/s12864-017-3811-6) contains supplementary material, which is available to authorized users.

## Background

miRNAs participate in the regulation of the expression of protein-coding genes at the post-transcriptional stage [1]. miRNAs, as a part of the RNA-induced silencing complex, bind to mRNAs and interfere with translation or promote mRNA destruction [2]. In the last two decades, properties of miRNAs and their influences on the expression of the genes involved in all key cellular processes have been established. The actions of miRNAs on the cell cycle [3], apoptosis [4], differentiation [5], and growth and development of plants [6] and animals [7] have been shown. Connections between miRNA expression and the development of various diseases have been established. miRNA concentrations change in cancer [8] and cardiovascular diseases [9]. Metabolic perturbations change miRNA concentrations in cells [10]. The aforementioned roles do not encompass all of the biological processes in which miRNAs participate, which further proves the importance of their biological functions. Despite the significant success in the study of miRNA properties, there are obstacles in identifying the target genes of miRNAs. Normally, one miRNA interacts with the mRNA of one gene. However, there are miRNAs that can bind to many mRNAs, and one mRNA can be the target of many miRNAs, which significantly complicates the study of the properties of miRNAs and their diagnostic and medical applications. There are more than 2,500 miRNAs in the human genome, and they are believed to act on 60% or more genes. Therefore, it is difficult to draw specific conclusions about the participation of miRNAs in specific biological processes, and until then the connections between the majority of miRNAs and their target genes will remain unknown. Recently, a set of unique miRNAs (umiRNA) were identified that have hundreds of target genes and bind to mRNAs with high affinity [11–14]. The binding sites of these umiRNAs are located in the 3’UTRs, CDSs, and 5’UTRs of mRNAs. Among these umiRNAs, miR-619-5p interacts with the largest number of target genes that have the greatest number of binding sites with complete complementarity of miR-619-5p and mRNAs. It is necessary to identify many miRNA binding sites in the mRNAs of these genes for the control of gene expression. Furthermore, it is important to control the expression of the corresponding gene complexes that are functionally associated with miRNAs. Therefore, we have studied a unique miR-619-5p that binds to the mRNAs of several hundred human and orthologous genes.

## Methods

The nucleotide sequences of mRNAs of human genes (*Homo sapience – Hsa*) and orthologous genes (*Bos mutus -* The wild yak *(Bmu), Callithrix jacchus –* The common marmoset *(Cja), Camelus dromedarius –* Arabian camel *(Cdr), Camelus ferus –* The wild Bactrian camel *(Cfe), Chlorocebus sabaeus –* The green monkey *(Csa), Colobus angolensis palliatus –* The Angola colobus *(Can), Equus caballus -* The horse *(Eca), Gorilla gorilla -* The western gorilla *(Ggo)*, *Macaca fascicularis –* The crab-eating macaque *(Mfa), Macaca mulatta –* The rhesus macaque *(Mmu)*, *Macaca nemestrina -* Pig-tailed macaque *(Mne), Mandrillus leucophaeus –* The drill (*Mle), Nomascus leucogenys -* The northern white-cheeked gibbon *(Nle), Ovis aries –* The sheep *(Oar), Pan paniscus -* Bonobos *(Ppa), Pan troglodytes –* The common chimpanzee *(Ptr), Papio anubis –* The olive baboon *(Pan), Pongo abelii -* The Sumatran orangutan *(Pab)*, *Rhinopithecus roxellana* – The golden snub-nosed monkey *(Rro)*) were downloaded from NCBI GenBank (http://www.ncbi.nlm.nih.gov) [15] in FASTA format using Lextractor002 script [11]. Nucleotide sequences of human mature miR-619-5p (GCUGGGAUUACAGGCAUGAGCC) were downloaded from the miRBase database (http://mirbase.org) [16]. The miR-619-5p binding sites in the 5’-untranslated regions (5’UTRs), the coding domain sequences (CDSs) and the 3’-untranslated regions (3’UTRs) of several genes were predicted using the MirTarget program [12]. This program defines the features of binding: a) the localization of miRNA binding sites in the 5’UTRs, the CDSs and the 3’UTRs of the mRNAs; b) the free energy of hybridization (∆G, kJ/mole). The ratio ΔG/ΔGm (%) was determined for each site (ΔGm equals the free energy of miRNA binding with its perfect complementary nucleotide sequence).

## Results

The search of 2,750 human microRNAs (miRNAs) binding sites in 12,175 mRNAs of human genes using the MirTarget program has been completed. The mRNAs have different miRNA binding site origins, lengths, quantities, and properties. The list of miR-619-5p target genes and the positions of binding sites are outlined in Table 1. miR-619-5p is 22 nucleotides in length and is coded by an intron of the slingshot protein phosphatase 1 (*SSH1*) gene, which is located on chromosome 12 [17, 18]. mRNAs of 201 genes have complete complementary binding sites for miR-619-5p (ΔG/ΔGm = 100%). Therefore, the energy of interaction of miR-619-5p with mRNA of all the genes listed in the table is the same and equal to ΔG = −121 kJ/mole.

**Table 1:** Positions of miR-619-5p binding sites and disease or function of target genes

Gene	Site, nt	Disease or function	PMID	Gene	Site, nt	Disease or function	PMID
*ACSL6*	4639	prostate cancer	19064571	*MRPS25*	1609	uncharacterized	26302410
*ADAL*	2041	proliferation	23645737	*MSH3*	4139	carcinogenesis	24934723
*ADAM17*	3466	breast cancer	22967992	*NANOS1*	3219	retinoblastoma	25100735
*AGMAT*	2207	renal carcinoma	14648699	*NCMAP*	2259	uncharacterized	
*AK1*	1449	hypertension	23863634	*NDUFAF7*	1697	leukemia	24292274
*AKT2*	4571	neuroblastoma	23468863	*NDUFC2*	1646	colon cancer	25804238
*ALDH3A2*	2617	detoxification	9829906	*NLN*	4215	Parkinson’s D.	25378390
*ANKRD16*	2165	breast cancer	20453838	*NRIP2*	2075	atopic asthma	17075290
*AP5B1*	4316	differentiation	15146197	*NSL1*	3063	kinetochore-protein	16585270
*ARGFX*	2642	development	20565723	*NXPE3*	7447	hepatocarcinoma	26883180
*ARHGEF39*	1307	tumorogenesis	22327280	*OPTN*	2332	glaucoma	26302410
*ARL11*	1033	tumorogenesis	18337727	*PAG1*	8156	prostatic cancer	21092590
*ATCAY*	2991	schizophrenia	19165527	*PAQR5*	4439	ovarian cancer	21761364
*ATP1A2*	4410	tumorogenesis	23474907	*PARK2*	3729	Parkinson’s D.	26860075
*BCL2L15*	2650	apoptosis	16690252	*PBLD*	2077	hepatocarcinoma	26594798
*BPNT1*	1128	ovarian cancer	20628624	*PCGF5*	5089	Alzheimer’s D.	16385451
*C15orf40*	523	uncharacterized		*PCSK5*	8613	tumorogenesis	21094132
*C17orf75*	2895	uncharacterized		*PDAP1*	1926	proliferation	23555679
*C17orf75*	3672			*PDCD4*	3221	tumorogenesis	26871813
*C21orf58*	2668	uncharacterized	11707072	*PEX2*	3056	cerebellar ataxia	21392394
*C4orf19*	2068	uncharacterized		*PGPEP1*	1476	liver cirrhosis	25687677
*C6orf170*	4113	uncharacterized	20159594	*PIK3R2*	3345	tumorogenesis	26677064
*C8orf44*	336**	uncharacterized		*PNPLA1*	1991	childhood obesity	19390624
*C8orf44*	1626			*PODNL1*	1876	uncharacterized	12477932
*C9orf85*	871	uncharacterized		*POFUT1*	4679	hepatocarcinoma	27003260
*CACNB2*	4301	hypertension	25966706	*POLH*	5550	ovarian cancer	25831546
*CACNG8*	3218	cardiomyopathy	26710323	*PPM1K*	2192	diabetes mellitus	23446828
*CACNG8*	5006			*PPP1R12B*	5156	childhood asthma	23640410
*CACNG8*	7535			*PRRG4*	998	Parkinson’s D	19772629
*CALHM1*	2896	Alzheimer’s D.	26944452	*PSMB2*	2925	proteolysis	21660142
*CCBE1*	3321	ovarian cancer	19935792	*PTCD3*	4116	osteosarcoma	19427859
*CCDC114*	261*	dyskinesia	23506398	*PTK6*	2233	tumorogenesis	27311570
*CD109*	6841	bladder cancer	20946523	*QRFPR*	1949	metabolic S.	16648250
*CD36*	4042	atherosclerosis	16515687	*RAB11FIP1*	4928	cell transport	26790954
*CD68*	1398	carcinomas	21113139	*RAB3IP*	3975	tumorogenesis	12007189
*CDAN1*	4296	erythropoiesis	19336738		7022		
*CDHR3*	4878	asthma	25848009	*RAB7L1*	1693	Parkinson’s D.	26914237
*CEP68*	4394	cervical cancer	17570516	*RBBP9*	1818	tumorogenesis	21933118
*CHST5*	2946	colon carcinoma	12107080	*RGS3*	205*	cardiovascular D.	24375609
*CHST6*	2979	dystrophy	20539220	*RPS6KA6*	7136	tumorogenesis	26732474
*CHST6*	3876			*SCN11A*	5871	neurophaty	25791876
*CIAO1*	2416	tumorogenesis	9556563	*SEPT11*	4033	hepatocarcinoma	20419844
*CIAO1*	3814			*SEPT14*	1575	Parkinson’s D	27115672
*CLEC19A*	1747	lectin	12975309	*SGTB*	3142	lymphopoesis	2158125
*CLTC*	7006	pancreatic cancer	23228632	*SH3GLB1*	4856	prostate cancer	27748942
*CORO2A*	2227	colon cancer	23490283	*SLC15A2*	4333	hepatocarcinoma	25965825
*COX18*	1264	tumorogenesis	20819778	*SLC17A5*	2389	cardiovascular D	27872510
*CPM*	2698	renal carcinoma	23172796	*SLC26A2*	5066	colorectal cancer	23840040
*CPM*	4996			*SLC26A4*	4210	hearing loss	27729126
*CPT2*	2557	sudden death	21641254	*SLC28A2*	2196	chronic hepatitis C	23195617
*CYB5RL*	3426	transcription	16344560	*SLC7A11*	6304	tumorogenesis	26729415
*CYP20A1*	2539	tumorogenesis	15191668	*SLC7A14*	8487	breast cancer	20379614
*CYP20A1*	4709			*SNX22*	902	liver-disease	21988832
*CYP27C1*	3823	self-rated health	20707712	*SOWAHC*	3417	retrotransposon	22234889
*CYP2W1*	2176	colorectal cancer	22993331	*SPATA13*	5020	colorectal cancer	17599059
*DAP3*	1842	breast cancer	22287761	*SPATA5*	5648	microcephaly	26299366
*DCAF10*	3305	lung cancer	28336923	*SPATS2*	3332	breast cancer	20379614
*DCAF10*	4559			*SPN*	5287	tumorogenesis	25551301
*DCLRE1C*	2966	Omenn syndrome	25981738	*STAC2*	2241	inherited ataxias	16713569
*DDOST*	1782	hyperglycemia	22305527	*SYNJ2BP*	1298	breast cancer	19349195
*DHODH*	1709	melanoma	21430780	*SYNJ2BP*	4175		
*DHRS9*	1281*	tumorogenesis	26254099	*TCEB1*	1964	tumorogenesis	23083832
*DNAL1*	4925	dyskinesia	15845866	*TIGD6*	3439	uncharacterized	
*DSCR6*	1706	Down syndrome	10814524	*TMEM156*	1593	uncharacterized	
*ERBB3*	5104	tumorogenesis	26689995	*TMEM19*	3510	uncharacterized	
*FADS6*	1777	liver disease	21988832,	*TMEM213*	875	uncharacterized	
*FAM161A*	2785	retinal disease	25749990	*TMEM214*	1190	uncharacterized	
*FAM227A*	4981	cancer	26759717	*TMEM50B*	1026	uncharacterized	
*FAM84B*	3626	tumorogenesis	25980316	*TMEM56*	1243	nicotine dependence	20379614
*FBLIM1*	2126	breast cancer,	23645746	*TMF1*	4736	prostate cancer	19330832
*FBXL22*	1411	cardiomyopathy	24324551	*TMOD2*	7816	bladder cancer	15095301
*FBXO27*	1535	leukemia	126433	*TNFRSF10A*	1621	cancer	27780136
*FGD4*	7619	cancer	22589722	*TNFRSF10D*	1532	cancer	26542757
*FKBP14*	1515	ovarian cancer	27931282	*TOP3A*	3814	leukaemia	22050635
*FKBP14*	2129			*TPRG1L*	1754	uncharacterized	
*FKBP5*	7114	schizophrenia	25522420	*TRIM72*	1885	ischemia	26790476
*FXN*	3288	metabolic disease	26717909	*TRPM7*	8079	neuroblastoma	27402209
*GDPD1*	1559	phosphodiesterase	18991142	*TRPM7*	8221	carcinoma	26779625
*GEMIN8*	2172	neuropathy	16434402	*TXNDC15*	2460	thrombosis	21642008
*GGT6*	1956	ovarian cancer	25356737	*TYW5*	3692	schizophrenia	23974872
*GK5*	3808	glioblastoma	25936394	*UACA*	6120	lung cancer	22407486
*GK5*	6355	glioblastoma	25936394	*UACA*	6120	thyroid diseases	15358194
*GLB1L*	2224	phosphatase	21382349	*UBIAD1*	2881	cancer	23759948
*GOLGA3*	7240	immune disease	17711851	*UBXN2A*	1665	colon cancer	24625977
*GP2*	1877	crohn disease	22891285	*UPK1B*	1513	cancer	16354592,
*GPR65*	3309	tumorogenesis	24152439	*UQCRB*	1269	colorectal cancer	22545919
*GPR65*	3309	immune diseases	15665078	*USP29*	2*	protease	10958632
*GPR82*	2664	uncharacterized		*VHL*	3764	tumorogenesis	27460078
*GPRIN2*	6676	schizophrenia	27244233	*VHL*	3898		
*GTPBP10*	1873	prostate cancer	27409348	*VWA2*	3366	colon cancer	15580307
*H6PD*	5754	tumorogenesis	15221007	*WDR73*	1736	microcephaly	25466283
*HM13*	1745	glioblastoma	28198167	*XIAP*	5681	ovarian cancer	26779627
*IFIT3*	1864	pancreatic cancer	25650658	*YAE1D1*	1548	oral cancer	23318452
*ISY1*	686**	uncharacterized		*ZBTB24*	4842	hepatocarcinoma	27730394
*IYD*	1658	hypothyroidism.	18765512	*ZC3H12D*	2812	Acute lung injury	26059755
*KIAA1456*	2536	colorectal cancer	24743840	*ZDHHC20*	3390	tumorogenesis	20334580
*KIF11*	3598	tumorogenesis	28011472	*ZFP30*	3463	hypertension	19851296
*KLHL23*	2570	tumorogenesis	23676014	*ZNF114*	1827	transcription factor	8467795
*KPNA1*	5711	breast cancer	26052702	*ZNF197*	3446	thyroid cancer	12682018
*KREMEN1*	2199	schizophrenia	20153141	*ZNF320*	5534	glioblastoma	11536051
*KREMEN1*	2792	schizophrenia	20153141	*ZNF429*	2081**	transcription factor	7865130
*LAX1*	2057	uncharacterized		*ZNF445*	8820	transcription factor	16368201
*LILRA6*	2201	tumorogenesis	26769854	*ZNF461*	3087	transcription factor	15004467
*LIMD1*	5735	breast cancer	27656835	*ZNF549*	3736	transcription factor	16344560
*LIMS1*	3931	cancer	27590440	*ZNF557*	4791	transcription factor	15851553
*LMOD3*	3224	myopathy	25250574	*ZNF626*	4620	liver diseases	18255255
*LMOD3*	3993	Alzheimer’s D	22881374	*ZNF667*	3240	transcription factor	17397802
*METTL6*	1188	breast cancer	25151356	*ZNF716*	2799	cardiovascular D	24376456
*MR1*	3664	hepatocarcinoma	26823810	*ZNF780B*	5415	transcription factor	15057824
*MREG*	1540	pulmonary D	20463177	*ZNF84*	4920	transcription factor	11856868
				*ZNF841*	3422	transcription factor	24280104

Notes: * - 5’UTR, **- CDS; others – 3’UTR, D - disease

The mRNAs of 201 human genes have complete complementary binding sites of miR-619-5p in the 3’UTR (214 sites), CDS (3 sites), and 5’UTR (4 sites). The mRNAs of 27 genes have four binding sites, seven genes have five binding sites, and *CATAD1, ICA1L*, *GK5*, *POLH,* and *PRR11* genes have six miR-619-5p binding sites. The mRNAs of *OPA3* and *CYP20A1* genes have eight and ten binding sites, respectively. All of these sites are located in the 3’UTRs of mRNAs.

The target genes of the miR-619-5p carry out one or more different functions and are involved in the development of various diseases (Table 1).

The mRNAs of the *C17orf75, C8orf44, CIAO1, CPM, CYP20A1, DCAF10, FKBP14, RAB3IP, SYNJ2BP, VHL* genes have two complete complementary binding sites for miR-619-5p, and the mRNA of the *CACNG8* gene has three such binding sites. This indicates a stronger dependence of the expression of these genes on miR-619-5p.

One of the methods to establish the credibility of the presence of miRNA binding site in the mRNA is to verify this site in the mRNAs of orthologous genes. In finding the miRNA binding sites raises the question of the level of reliability of the found sites. One effective way to establish the credibility of the binding sites is to establish binding sites in the orthologous genes and the identification of orthologous miRNA. Location of binding site in the protein coding region facilitates its conservation in evolution, especially if the corresponding oligopeptide plays an important role in the function of the protein. miR-619-5p binding sites with complete complementarity (ΔG/ΔGm is 100%) to the mRNAs of the four genes are located in the 5’UTRs (Table 2).

**Table 2:** Variation of positions and nucleotide sequences of miR-619-5p binding sites in the 5’UTRs of mRNAs of mammal genes

Species	Gene	Position of site, nt	Nucleotide sequence
*Hsa*	*CCDC114*	261	GCAUGCU**GGCUCAUGCCUGUAAUCCCAGC**ACUUUGG
*Hsa*	*DHRS9*	1281	GCGCGGU**GGCUCAUGCCUGUAAUCCCAGC**ACUUUGG
*Hsa*	*RGS3*	205	GCGCAGU**GGCUCAUGCCUGUAAUCCCAGC**ACUUUGG
*Ptr*	*RGS3*	1	GCGCAGU**GGCUCAUGCCUGUAAUCCCAGC**ACUUUGG
*Nle*	*RGS3*	205	GCACGGU**GGCUCAUGCCUGUAAUCCCAGC**ACUUUGG
*Hsa*	*USP29*	2	CUGGCCA**GGCUCAUGCCUGUAAUCCCAGC**ACUUUGG
*Pab*	*USP29*	52	CUGGCCA**GGCUCAUGCCUGUAAUCCCAGC**ACUUUGG
*Nle*	*USP29*	52	CUGGCCA**GGCUCAUGCCUGUAAUCCCAGC**ACUUUGG
*Mle*	*USP29*	47	CUGGCCA**GGCUCAUGCCUGUAAUCCCAGC**ACUUUGG
*Can*	*USP29*	98	CUGGCCA**GGCUCAUGCCUGUAAUCCCAGC**AUUUUGG
*Ggo*	*USP29*	100	CUGGCCA**GGCUCAUGCCUGUAAUUCCAGC**ACUUUGG
*Rro*	*USP29*	52	CUGGCCA**GGCUCAUGCCUGUAAUCGCAGC**ACUUUGG

Notes: In the table 2-5 the bold type indicates the binding site of miR-619-5p

Before the 5’ end and after the 3’ end of miR-619-5p binding site, nucleotides are not homologous. The mRNAs of *RGS3* and *USP29* orthologous genes have binding sites in *H. sapiens*, *N. leucogenys*, *P. abelii*, *M. leucophaeus*, *C. angolensis palliatus*, *G. gorilla,* and *R. roxellana*.

miR-619-5p has two binding sites in the 5’UTRs of mRNAs of *ANAPC16*, *CYB5D2,* and *PRR5* and three binding sites in the mRNA of *DNASE1*.

mRNAs of some genes have binding sites for miR-619-5p within their 5’UTRs and 3’UTRs or CDSs and 3’UTRs. For example, *ATAD3C, C14orf182,* and *CYB5RL* have miR-619-5p binding sites in the 5’UTRs and 3’UTRs, and *C8orf44*, *ISY1,* and *ZNF714* have miR-619-5p binding sites in the CDSs and 3’UTRs.

The nucleotide sequences of miR-619-5p binding sites are located in the CDSs of the *C8orf44*, *C8H8orf44, ISY1, ZNF429,* and *ZNF714* genes and encode the following oligopeptides (Table 3).

**Table 3:** Variation of amino acid sequences coding in miR-619-5p binding sites in the mRNAs of orthologous genes

Species	Gene	Amino acid sequence
*Hsa*	*C8orf44*	HWKGRAR**WLMPVIP**ALWEAKA
*Hsa*	*C8H8orf44*	HWKGRAR**WLMPVIP**ALWEAKA
*Pab*	*C8H8orf44*	HWKGWAR**WLTPVIP**ALWEAKA
*Pan*	*C8H8orf44*	HWKGRAR**WLMPAIP**ALWEAKX
*Ppa*	*C8H8orf44*	HWKGRAQ**WLTPVIP**ALWEAKA
*Ptr*	*C8H8orf44*	HWKGRAQ**WLTPVIP**ALWEAKA
*Hsa*	*ISY1*	EKERQVR**WLMPVIP**ALWEAEA
*Hsa*	*ZNF714*	KIQQGMV**AHACNPN**TLRGLGE
*Ggo*	*ZNF714*	KIQQGMV**AHACNPN**TLRGLGE
*Ptr*	*ZNF714*	KIQQGMV**AHACNPN**TXRGLGE
*Ppa*	*ZNF714*	KIQQGMV**AHACNPN**TLRGLGE
*Hsa*	*ZNF429*	IHRMGVV**AHACNPS**TLGGRGG
*Mfa*	*ZNF429*	IHRLGVV**AHACNPS**TLGGRGG
*Mmu*	*ZNF429*	IHRLGVV**AHACNPS**TLGGRGG
*Mne*	*ZNF429*	IHRLGVV**AHACNPS**TLGGRGG

*C8H8orf44, C8orf44,* and *ISY1* genes encode the WLMPVIP oligopeptide, which is also present in the orthologous proteins of *P. abelii*, *P. anubis*, *P. paniscus*, and *P. troglodytes*. The mRNA of transcription factor *ZNF429* and *ZNF429* genes binding sites are encoded the AHACNP oligopeptide in the another reading frame. The first two oligopeptides are encoded in one open reading frame (ORF) and the amino acid sequences are highly conserved. The homologous oligonucleotide of the miR-619-5p binding site in the mRNA of *ZNF714* gene codes for an oligopeptide in a different ORF.

The presence of miR-619-5p binding sites in the CDSs of five genes with different functions and the evolutionary conservation of these sites signify the role of miRNA in the regulation of the expression of these genes. The nucleotide sequences of specific regions of mRNAs of *C8H8orf44, C8orf44*, *ISY1, ZNF429,* and *ZNF714* genes that contain miR-619-5p binding sites in the CDSs are homologous among themselves and to the binding sites located in the 5’UTRs and 3’UTRs.

The miRNA binding sites in the coding region, as opposed to the 3’UTR and 5’UTR, clearly demonstrate the relationship between miRNA and mRNA by their conserved amino acid sequences in orthologous proteins. miRNA binding site can be translated by two open reading frames that encode WLTPVIPA and AHACNPS oligopeptides. In the third reading frame, the miR-619-5p binding site has a stop codon. However, in the genes studied, no such sequence was found. In the absence of complete complementarity between miR-619-5p and its binding site, miR-619-5p uses a site containing the corresponding mutation in the CDS for the regulation of gene expression. Thus, a single miRNA binding site in the mRNA of various genes may correspond to three different oligopeptides. Generally, one out of these three oligopeptides is present in the proteins encoded by the orthologous genes.

*ISY1* orthologous genes in *H. sapiens*, *P. troglodytes*, and *N. leucogenys* encode a protein containing QVRWLMPVIPALWEAEAGGSQA oligopeptide sequence (Table 4).

**Table 4:** Amino acid sequences coding in miR-619-5p binding sites in the mRNA of *ISY1* gene of orthologous genes

Species	Amino acid sequence
*Hsa*	PGVRELFEKERQVR**WLMPVIP**ALWEAEAGGSQALPPPRKTRAELMKA
*Ptr*	PGVRELFEKERQVR**WLMPVIP**ALWEAEAGGSQALPPPRKTRAELMKA
*Nle*	PGVRELFEKERQAR**WLTPVIP**ALWEAEAGGSQALPPPRKTRAELMKA
Hsa*	PGVRELFEKEP----------------------LPPPRKTRAELMKA
*Bmu*	PGVRELFEKEP----------------------LPPPRKTRAELMKA
*Cdr*	PGVRELFEKEP----------------------LPPPRKTRAELMKA
*Cfa*	PGVRELFEKEP----------------------LPPPRKTRAELMKA
*Cja*	PGVRELFEKEP----------------------LPPPRKTRAELMKA
*Eca*	PGVRELFEKEP----------------------LPPPRKTRAELMKA
*Ggg*	PGVRELFEKEP----------------------LPPPRKTRAELMKA
*Mmu*	PGVRELFEKEP----------------------LPPPRKTRAELMKA
*Nle*	PGVRELFEKEP----------------------LPPPRKTRAELMKA
*Oar*	PGVRELFEKEP----------------------LPPPRKTRAELMKA
*Pab*	PGVRELFEKEP----------------------LPPPRKTRAELMKA
*Ppa*	PGVRELFEKEP----------------------LPPPRKTRAELMKA
*Rro*	PGVRELFEKEP----------------------LPPPRKTRAELMKA

* *RAB43* - human *ISY1* paralog gene

However, the *RAB43* gene, which is paralogous to human *ISY1,* lacks the nucleotide sequence encoding the QVRWLMPVIPALWEAEAGGSQA oligopeptide. Additionally, *ISY1* gene in the genomes of other animals also lacks the nucleotide sequence encoding this oligopeptide (Table 4).

Nucleotide sequences of miR-619-5p binding sites in the mRNAs of *ADAM17, ALDH3A2,* and *ARL11* orthologous genes are shown in Table 5.

**Table 5:** Variation of nucleotide sequences of miR-619-5p binding sites in the 3’UTR of mRNAs of *ADAM17, ALDH3A2,* and *ARL11* of orthologs

Species	Gene	Position, nt	Nucleotide sequence
*Hsa*	*ADAM17*	3466	TGGGAGTGGT**GGCTCATGCCTGTAATCCCAGC**ACTTGGAGAGG
*Cat*	*ADAM17*	3485	GGGGCGCAGT**GGCTCATGCCTGTAATCCCAGC**ACTTTGGGAGG
*Mmul*	*ADAM17*	3491	GGGGCGCGGT**GGCTCATGCCTGTAATCCCAGC**ACTTTGGGAGG
*Mne*	*ADAM17*	3438	GGGGCGCGGT**GGCTCATGCCTGTAATCCCAGC**ACTTTGGGAGG
*Ptr*	*ADAM17*	3449	TGGGAGTGGT**GGCTCATGCCTGTAATCCCAGC**ACTTGGAGAGG
*Rro*	*ADAM17*	3425	GGGGCGCGGT**GGCTCATGCCTGTAATCCCAGC**ACTTTGGGAGG
*Hsa*	*ALDH3A2*	2617	CGGGCGTGGT**GGCTCATGCCTGTAATCCCAGC**ACTTTGGGAGG
*Cja*	*ALDH3A2*	3444	CGGGCGTGGT**GGCTCATGCCTGTAATCCCAGC**ACTTTAGGAGG
*Ggo*	*ALDH3A2*	2712	CGGGCGTGGT**GGCTCATGCCTGTAATCCCAGC**ACTTTGGGAGG
*Mmul*	*ALDH3A2*	2509	CGGACATGGT**GGCTCATGCCTGTAATCCCAGC**ACTTTGGGAGG
*Mne*	*ALDH3A2*	2504	CGGACATGGT**GGCTCATGCCTGTAATCCCAGC**ACTTTGGGAGG
*Nle*	*ALDH3A2*	2714	TGGTCATGGT**GGCTCATGCCTGTAATCCCAGC**ACTTTGGGAGG
*Pab*	*ALDH3A2*	2297	TGGGCATGGT**GGCTCATGCCTGTAATCCCAGC**ACTTTGGGAGG
*Ppa*	*ALDH3A2*	2715	CGGGCATGGT**GGCTCATGTCTGTAATCCCAGC**ACTTTGGGAGG
*Ptr*	*ALDH3A2*	2711	CGGGCATGGT**GGCTCATGTCTGTAATCCCAGC**ACTTTGGGAGG
*Rro*	*ALDH3A2*	2727	CGGACGTGGT**GGCTCATGCCTGTAATCCCAGC**ACTTTGGGAGG
*Hsa*	*ARL11*	1033	TTGGCCCGGT**GGCTCATGCCTGTAATCCCAGC**ACTGTGGGAGA
*Cat*	*ARL11*	1642	CAGATGCAGT**GGCTCATGCCTGTAATCCCAGC**ACTTTGGGTGG
*Mfa*	*ARL11*	1698	CAGATGCAGT**GGCTCATGCCTGTAATCCCAGC**ACTTTGGGTGG
*Mmul*	*ARL11*	1747	CAGATGCAGT**GGCTCATGCCTGTAATCCCAGC**ACTTTGGGTGG
*Mne*	*ARL11*	1024	TTGGCACGGT**GGCTCATGCCTGTAATCCCAGC**ACTTTGGGAGA
*Mne*	*ARL11*	1471	CAGATGCAGT**GGCTCATGCCTGTAATCCCAGC**ACTTTGGGTGG
*Ptr*	*ARL11*	1353	CGGGCATGGT**GGCTCATGTCTGTAATCCCAGC**ACTTTGGGAGG
*Rro*	*ARL11*	1254	CAGGTGCAGT**GGCTCATGCCTGTAATCCCAGC**ACTTTGGGCGG

These orthologous genes are characterized by highly conserved nucleotide sequence GGCTCATGCCTGTAATCCCAGC of miR-619-5p binding sites. This shows that the interaction of miR-619-5p with mRNAs of these genes is conserved during evolution. Some of the human miR-619-5p target genes and their corresponding orthologous genes have two miR-619-5p binding sites in their mRNAs.

Table 6 shows the nucleotide sequences of two miR-619-5p binding sites in the 3’UTR of mRNAs of *ERBB3, FBLIM1,* and *FKBP14* orthologous genes.

**Table 6:** Variation of nucleotide sequences of two miR-619-5p binding sites in the 3’UTR of mRNAs of *ERBB3, FBLIM1,* and *FKBP14* of orthologs

Species	Gene	Position, nt	Nucleotide sequence
*Hsa*	*ERBB3*	4950	CGGGCATGGT**GGCTCATGCCTGTAATCTCAGC**ACTTTGGGAG
*Hsa*	*ERBB3*	5104	TGGGTGCAGT**GGCTCATGCCTGTAATCCCAGC**CAGCACTTTG
*Csa*	*ERBB3*	4989	CGGGCATGGT**GGCTCATGCCTGTAATCCTAGC**ACTTTGGGAG
*Csa*	*ERBB3*	5149	TGGGCGCTGT**GGCTCATGCCTGCAATCCCAGC**ACTTTGGGAG
*Mfa*	*ERBB3*	5114	TGGGCATGGT**GGCTCATGCCTGTAATCCCAGC**ACTTTGGGAG
*Mfa*	*ERBB3*	5269	TGGGCGCTGT**GGCTCATGCCTGCAATCCCAGC**CCTTTGGGAG
*Mmu*	*ERBB3*	5114	TGGGCATGGT**GGCTCATGCCTGTAATCCCAGC**ACTTTGGGAG
*Mmu*	*ERBB3*	5269	TGGGCGCTGT**GGCTCATGCCTGCAATCCCAGC**CCTTTGGGAG
*Mne*	*ERBB3*	5112	CGGGCATGGT**GGCTCATGCCTGTAATCCCAGC**ACTTTGGGAG
*Mne*	*ERBB3*	5267	TGGGCGCTGT**GGCTCATGCCTGCAATCCCAGC**CCTTTGGGAG
*Pan*	*ERBB3*	5106	CGGGCATGGT**GGCTCATGCCTGTAATCCCAGC**ACTTTGGGAG
*Pan*	*ERBB3*	5274	TGGGCGCTGT**GGCTCATGCCTGCAGTCCCAGC**ACTTTGGGAG
*Ptr*	*ERBB3*	5105	CGGGCATGGT**GGCTCATGCCTGTAATCTCAGC**ACTTTGGGAG
*Ptr*	*ERBB3*	5243	TGGGTGCAGT**GGCTCATGCCTGTAATCCCAGC**CAGCACTTTG
*Mne*	*FBLIM1*	1938	TGGGCGTGGT**GGCTCATGCCTGTAATCCCTGC**ACTTTGGGAG
*Mne*	*FBLIM1*	5267	TGGGCGCTGT**GGCTCATGCCTGCAATCCCAGC**CCTTTGGGAG
*Pab*	*FKBP14*	1514	CAGGCACGGT**GGCTCACGCCTGTAATCCCAGC**ACTTCGGGAG
*Pab*	*FKBP14*	2128	TGGGTGTGGT**GGCTCATGCCTGTAATCCCAGC**ACTTTGGGGG

Notes: The black type indicates the binding site of miR-619-5p

Table 7 shows the degree of conservation of miR-619-5p binding sites in the 201 mRNAs of target genes. All mRNAs with complete complementarity to miR-619-5p binding sites (ΔG/ΔGm is 100%) were divided into four groups, and the frequency of occurrence of nucleotides was determined in each group. The results suggest that miR-619-5p binding sites are highly conserved. The binding site GGCTCATGCCTGTAATCCCAGC does not change and in each of the four gene groups the observed variability of nucleotides on the right and left is high.

**Table 7:**
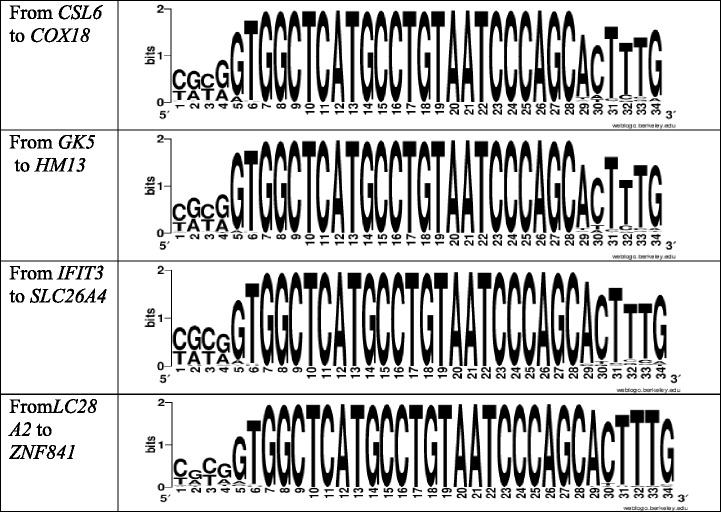
Variation of nucleotide sequences of mRNA region with miR-619-5p binding sites (See Additional file 1, 2, 3 and 4)

## Discussion

Here we have identified many miRNAs binding sites in the mRNAs of 201 human genes which indicates that umiRNAs act as coordinators of gene expression by participating in many biological processes. Previous studies have shown the influences of miRNAs on the expression of genes that encode the transcription factors [19, 20] and on the expression of proteins that participate in the cellular cycle [3, 21–23], apoptosis [4, 24–26], and stress responses [27]. It was shown the role of the mir-619-5p in the regulation of different pathological processes [28]. It was investigated the correlations between the expression of *MALAT1* and miR-619-5p, in addition to the association between the clinicopathological features and survival outcomes of patients with stage II and III colorectal cancer tumors [28]. It was observed, that hsa-miR-619-5p and hsa-miR-1184 microRNA expression significantly increased in prostatic cancer. MicroRNA-gene-net analysis indicated that miR-619-5p and other some miRNAs had the most important and extensive regulatory function for Qi-stagnation syndromes and Qi-deficiency syndromes in coronary heart disease [29].

One or several umiRNAs regulating the expression of hundreds of genes can create a system of interconnected processes in cells and organisms. Such role of these umiRNAs is possible because they circulate in the blood and have access to nearly all cells of an organism [30–32]. Our results provide the basis for studying the systemic roles of unique and normal miRNAs in the regulation of gene expression in human cells. The expression of many target genes is regulated by umiRNAs does not allow individual mRNAs of target genes to be expressed in more degree than others. The greater expression of one mRNA, the larger number of umiRNAs bind to this mRNA. This allows one umiRNA to maintain a certain balance of expression of the corresponding target genes. If umiRNA expression changes, such system is vulnerable. This will cause the development of pathology in the cell, tissue or body.

## Conclusions

The majority of miR-619-5p binding sites are located in the 3’UTRs of mRNAs of target genes. Some genes have miRNA binding sites in the 5’UTRs of mRNAs. It is necessary to maintain nucleotide sequences of the binding site of umiRNA in the CDSs of several genes. Different genes have binding sites for miRNAs that are read in different open reading frames. Therefore, identical nucleotide sequences encode different amino acids in different proteins. In encoded proteins, these sites encode conservative oligopeptides. The binding sites of miR-619-5p in 3’UTRs, 5’UTRs and CDSs are conservative in the orthologous mammalian genes.

## Additional files

Additional file 1: Figure S1. Variation of nucleotide sequences of mRNA region with miR-619-5p binding sites of genes from *CSL6* to *COX18* (Conservative binding sites are in bold) (PDF 218 kb)

Additional file 2: Figure S2. Variation of nucleotide sequences of mRNA region with miR-619-5p binding sites of genes from *GK5* to *HM13* (Conservative binding sites are in bold) (PDF 106 kb)

Additional file 3: Figure S3. Variation of nucleotide sequences of mRNA region with miR-619-5p binding sites of genes from *IFIT3* to *SLC26A4* (Conservative binding sites are in bold) (PDF 139 kb)

Additional file 4: Figure S4. Variation of nucleotide sequences of mRNA region with miR-619-5p binding sites of genes from *LC28A2* to *ZNF841* (Conservative binding sites are in bold). The data given in the Additional files 1, 2, 3 and 4 demonstrate the variability of the nucleotides before and after the binding sites of miR-619-5p, which is shown in the Weblogo schemes in the table 8. (PDF 151 kb)

## Abbreviations

CDSs: Coding domain sequences
miRNAs: Micrornas
ORF: Open reading frame
Umirna: Unique miRNA

## References

[1] Doxakis E (2013). Principles of miRNA-Target Regulation in Metazoan Models. Int J Mol Sci.

[2] Tang G (2005). siRNA and miRNA: an insight into RISCs. Trends Biochem Sci.

[3] Luo Q, Li X, Li J, Kong X, Zhang J, Chen L, Huang Y, Fang L (2013). MiR-15a is underexpressed and inhibits the cell cycle by targeting CCNE1 in breast cancer. Int J Oncol.

[4] Li X, Chen YT, Josson S, Mukhopadhyay NK, Kim J, Freeman MR, Huang WC (2013). MicroRNA-185 and 342 inhibit tumorigenicity and induce apoptosis through blockade of the SREBP metabolic pathway in prostate cancer cells. PLoS One.

[5] Qian NS, Liu WH, Lv WP, Xiang X, Su M, Raut V, Chen YL, Dong JH (2013). Upregulated MicroRNA-92b regulates the differentiation and proliferation of EpCAM-positive fetal liver cells by targeting C/EBPß. PLoS One.

[6] Poethig RS (2013). Vegetative phase change and shoot maturation in plants. Curr Top Dev Biol.

[7] Ling YH, Ding JP, Zhang XD, Wang LJ, Zhang YH, Li YS, Zhang ZJ, Zhang XR (2013). Characterization of microRNAs from goat (Capra hircus) by Solexa deep-sequencing technology. Genet Mol Res.

[8] Knyazev EN, Fomicheva KA, Mikhailenko DS (2016). Plasma Levels of hsa-miR-619-5p and hsa-miR-1184 Differ in Prostatic Benign Hyperplasia and Cancer. Bull Exp Biol Med.

[9] Hou J, Wang J, Lin C, Fu J, Ren J, Li L, Guo H, Han X, Liu J (2014). Circulating MicroRNA Profiles Differ between Qi-Stagnation and Qi-Deficiency in Coronary Heart Disease Patients with Blood Stasis Syndrome. Evid Based Complement Alternat Med.

[10] Swaminathan S, Suzuki K, Seddiki N, Kaplan W, Cowley MJ, Hood CL (2012). Differential regulation of the Let-7 family of microRNAs in CD4+ T cells alters IL-10 expression. J Immunol.

[11] Ivashchenko A, Berillo O, Pyrkova A, Niyazova R, Atambayeva S (2014). MiR-3960 binding sites with mRNA of human genes. Bioinformation.

[12] Ivashchenko A, Berillo O, Pyrkova A, Niyazova R, Atambayeva S (2014). The properties of binding sites of miR-619-5p, miR-5095, miR-5096 and miR-5585-3p in the mRNAs of human genes. Biomed Res Int.

[13] Ivashchenko A, Berillo O, Pyrkova A, Niyazova R (2014). Binding Sites of miR-1273 Family on the mRNA of Target Genes. Biomed Res Int.

[14] Ivashchenko A, Berillo O, Pyrkova A, Niyazova R, Atambayeva S (2014). The binding sites of unique miRNAs in the human mRNAs. J Biotechnol.

[15] National Center for Biotechnology Information. http://www.ncbi.nlm.nih.gov

[16] Griffiths-Jones S, Grocock R, van Dongen S, Bateman A, Enright A (2006). miRBase: microRNA sequences, targets and gene nomenclature. Nucleic Acids Res.

[17] Cummins JM, He Y, Leary RJ, Pagliarini R, Diaz LA, Sjoblom T, Barad O, Bentwich Z, Szafranska AE, Labourier E, Raymond CK, Roberts BS, Juhl H, Kinzler KW, Vogelstein B, Velculescu VE (2006). The colorectal microRNAome. Proc Natl Acad Sci U S A.

[18] Ple H, Landry P, Benham A, Coarfa C, Gunaratne PH, Provost P (2012). The repertoire and features of human platelet microRNAs. PLoS One.

[19] Cui Q, Yu Z, Pan Y, Purisima EO, Wang E (2007). MicroRNAs preferentially target the genes with high transcriptional regulation complexity. Biochem Biophys Res Commun.

[20] Yan L, Kang M, Qin Z, Zhang W, Li Y, Ou H (2013). An intronic miRNA regulates expression of the human endothelial nitric oxide synthase gene and proliferation of endothelial cells by a mechanism related to the transcription factor SP-1. PLoS One.

[21] Wang P, Zou F, Zhang X, Li H, Dulak A, Tomko RJ, Lazo JS, Wang Z, Zhang L, Yu J (2013). microRNA-21 negatively regulates Cdc25A and cell cycle progression in colon cancer cells. Cancer Res.

[22] Cui X, Witalison EE, Chumanevich AP, Chumanevich AA, Poudyal D (2013). The induction of microRNA-16 in colon cancer cells by protein arginine deiminase inhibition causes a p53-dependent cell cycle arrest. PLoS One.

[23] Wang Y, Zheng X, Zhang Z, Zhou J, Zhao G (2012). MicroRNA-149 inhibits proliferation and cell cycle progression through the targeting of ZBTB2 in human gastric cancer. PLoS One.

[24] Wang Y, Lee CG (2009). MicroRNA and cancer focus on apoptosis. J Cell Mol Med.

[25] Li C, Hashimi SM, Good DA, Cao S, Duan W, Plummer PN, Mellick AS, Wei MQ (2012). Apoptosis and microRNA aberrations in cancer. Clin Exp Pharmacol Physiol.

[26] Lima RT, Busacca S, Almeida GM, Gaudino G, Fennell DA, Vasconcelos MH (2011). MicroRNA regulation of core apoptosis pathways in cancer. Eur J Cancer.

[27] Cawley K, Logue SE, Gorman AM, Zeng Q, Patterson J, Gupta S, Samali A (2013). Disruption of microRNA biogenesis confers resistance to ER stress-induced cell death upstream of the mitochondrion. PLoS One.

[28] Qiu G, Zhang X, Zhang S, Liu P, Wu W, Zhang J, Dai S (2016). Dysregulation of MALAT1 and miR-619-5p as a prognostic indicator in advanced colorectal carcinoma. Oncol Lett.

[29] Hou J, Wang J, Lin C, Fu J, Ren J, Li L, Guo H, Han X, Liu J (2014). Circulating MicroRNA Profiles Differ between Qi-Stagnation and Qi-Deficiency in Coronary Heart Disease Patients with Blood Stasis Syndrome. Hindawi Publishing Corporation Evidence-Based Complementary and Alternative Medicine.

[30] Kumar S, Keerthana R, Pazhanimuthu A, Perumal P (2013). Overexpression of circulating miRNA-21 and miRNA-146a in plasma samples of breast cancer patients. Indian J Biochem Biophys.

[31] Smith-Vikos T, Slack FJ (2013). MicroRNAs circulate around Alzheimer’s disease. Genome Biol.

[32] Reshmi G, Chandra SS, Babu VJ, Babu PS, Santhi WS, Ramachandran S, Lakshmi S, Nair AS, Pillai MR (2011). Identification and analysis of novel microRNAs from fragile sites of human cervical cancer: computational and experimental approach. Genomics.

