# Supplementary material for: The Binding Sites of miR-619-5p in the mRNAs of Human and Orthologous Genes
Source: BMC Genomics. 2017 Jun 1;18:428. doi: 10.1186/s12864-017-3811-6 (PMC5452331; doi:10.1186/s12864-017-3811-6)
Supplement: Supplementary file 1 — Variation of nucleotide sequences of mRNA region with miR-619-5p binding sites of genes from CSL6 to COX18 (Conservative binding sites are in bold) (PDF 218 kb) [file 12864_2017_3811_MOESM1_ESM.pdf]

Figure 1 Variation of nucleotide sequences of mRNA region with miR-619-5p binding sites of genes from *CSL6* to *COX18* (Conservative binding sites are in bold)

TGGGCGCGGT***GGCTCATGCCTGTAATCCCAGC***ACTTTGGGAG ACSL6 4639
CGGATGTGAT***GGCTCATGCCTGTAATCCCAGC***ACTTTGGGAA ADAL 2041
TGGGAGTGGT***GGCTCATGCCTGTAATCCCAGC***ACTTGGAGAG ADAM17 3466
TGGACACGGT***GGCTCATGCCTGTAATCCCAGC***ACTTTGGGGG AGMAT 2207
CCAATGAGGT***GGCTCATGCCTGTAATCCCAGC***ACTTTGGGAA AK1 1449
TGGGCACAGT***GGCTCATGCCTGTAATCCCAGC***AATTTGGGAG AKT2 4571
CGGGCGTGGT***GGCTCATGCCTGTAATCCCAGC***ACTTTGGGAG ALDH3A2 2617
CGGTTGCGGT***GGCTCATGCCTGTAATCCCAGC***ACTTTGGGAG ANKRD16 2165
TGGGTGCGGT***GGCTCATGCCTGTAATCCCAGC***ACTTTGAGAG AP5B1 4316
TGGGGGTGGT***GGCTCATGCCTGTAATCCCAGC***ACTTTGGGAG ARGFX 2642
CAGGCACAGT***GGCTCATGCCTGTAATCCCAGC***ACTTTGGGAA ARHGEF39 1307
TTGGCCCGGT***GGCTCATGCCTGTAATCCCAGC***ACTGTGGGAG ARL11 1033
AGGGCACAGT***GGCTCATGCCTGTAATCCCAGC***ACTCTGGGAG ATCAY 2991
TGGGTGCGGT***GGCTCATGCCTGTAATCCCAGC***ACTTTGGGAG ATP1A2 4410
CTGGCGCAGT***GGCTCATGCCTGTAATCCCAGC***ACTTTGGGAG BCL2L15 2650
CGGGCGCGGT***GGCTCATGCCTGTAATCCCAGC***ACTTTGGGAG BPNT1 1128
CGGGCGCAGT***GGCTCATGCCTGTAATCCCAGC***ACTTTGGGAG C15orf40 523
TGGGCACGAT***GGCTCATGCCTGTAATCCCAGC***ACTTTGAGAG C17orf75 2895
TGGGTGTGGT***GGCTCATGCCTGTAATCCCAGC***ACTTTGGGAG C17orf75 3672
AGGGGTGCAT***GGCTCATGCCTGTAATCCCAGC***ACTTTAAGAG C21orf58 2668
CGGGCGTGGT***GGCTCATGCCTGTAATCCCAGC***ACTTTGGGAG C4orf19 2068
CAGGCGCGGT***GGCTCATGCCTGTAATCCCAGC***ACTTTGGGAG C6orf170 4113
CAGGTGTGAT***GGCTCATGCCTGTAATCCCAGC***ACTTTGGGAG C8orf44 1626
TGGGCGTGGT***GGCTCATGCCTGTAATCCCAGC***TACTCAGGAG C9orf85 871
CGGGCGCAGT***GGCTCATGCCTGTAATCCCAGC***AATTTGGGAG CACNB2 4301
CGGGCGCGGT***GGCTCATGCCTGTAATCCCAGC***GCTTTGGGAG CACN8 3218
CGGGCGCAGT***GGCTCATGCCTGTAATCCCAGC***ACTTTGGGAG CACN8 5006
CAGGCTTGGG***GGCTCATGCCTGTAATCCCAGC***ACTCTGGGAG CACN8 7535
CAGGCGGAGT***GGCTCATGCCTGTAATCCCAGC***ACTTGGGAGG CALHM1 2896
CGGGCTTGGT***GGCTCATGCCTGTAATCCCAGC***ACTTTGGGAG CCBE1 3321
CAGGCGCAGT***GGCTCATGCCTGTAATCCCAGC***ACTCTGGGAG CD109 6841
CAGGCATGGT***GGCTCATGCCTGTAATCCCAGC***ACTTTGGGAG CD36 4042
CGGGCATGAC***GGCTCATGCCTGTAATCCCAGC***ACTTTGGGAG CD68 1398
CGGGTCTGGT***GGCTCATGCCTGTAATCCCAGC***ACTTTGAGAG CDAN1 4296
CGGGTGAGGT***GGCTCATGCCTGTAATCCCAGC***ACTCTGGGAG CDHR3 4878
TGGGCACGGT***GGCTCATGCCTGTAATCCCAGC***ACTTTGGGAG CEP68 4394
CGGGTGCGGT***GGCTCATGCCTGTAATCCCAGC***ATTTTGAGAG CHST5 2946
TGGATGCAGT***GGCTCATGCCTGTAATCCCAGC***ACTGTGGGAG CHST6 2979
CAGGCGTGGT***GGCTCATGCCTGTAATCCCAGC***ATTTTGGAAG CHST6 3876
CCAGCGCAGT***GGCTCATGCCTGTAATCCCAGC***ACTTTGGGAG CIAO1 2416
TGGGCATGGT***GGCTCATGCCTGTAATCCCAGC***ACTCTGGGAG CIAO1 3814
CAGGTGTAGT***GGCTCATGCCTGTAATCCCAGC***ACTTTGGGAA CLEC19A 1747
CAGGTGTGGT***GGCTCATGCCTGTAATCCCAGC***ACTTTGGGAG CLTC 7006
CAGACGCAGT***GGCTCATGCCTGTAATCCCAGC***ACTCTGGGAG CORO2A 2227
CAGGCACAGT***GGCTCATGCCTGTAATCCCAGC***ACTTTGGGAG COX18 1264
CAGGCATGGT***GGCTCATGCCTGTAATCCCAGC***ATTTTGGGAG CPM 2698
CAGGCACGGT***GGCTCATGCCTGTAATCCCAGC***ACTTTGGGAG CPM 4996
CGGGCATGGT***GGCTCATGCCTGTAATCCCAGC***ATTTTGAGAG CPT2 2557
CGGACACGGT***GGCTCATGCCTGTAATCCCAGC***ACTTTGGGAG CYP5RL 3426
CGGGAGTGGT***GGCTCATGCCTGTAATCCCAGC***ACTTTGGGAT CYP20A1 2539
CGGGCGTGAT***GGCTCATGCCTGTAATCCCAGC***ACTTTGGGAG CYP20A1 4709
CAGGCGAGGT***GGCTCATGCCTGTAATCCCAGC***ACTTTGGGAG CYP27C1 3823
TGGGCACAGT***GGCTCATGCCTGTAATCCCAGC***ACTTTGGAAG DAP3 1842
