# Supplementary material for: The Binding Sites of miR-619-5p in the mRNAs of Human and Orthologous Genes
Source: BMC Genomics. 2017 Jun 1;18:428. doi: 10.1186/s12864-017-3811-6 (PMC5452331; doi:10.1186/s12864-017-3811-6)
Supplement: Supplementary file 2 — Variation of nucleotide sequences of mRNA region with miR-619-5p binding sites of genes from GK5 to HM13 (Conservative binding sites are in bold) (PDF 106 kb) [file 12864_2017_3811_MOESM2_ESM.pdf]

Figure 2 Variation of nucleotide sequences of mRNA region with miR-619-5p binding sites of genes from *GK5* to *HM13* (Conservative binding sites are in bold)

CAGGCGCAGT***GGCTCATGCCTGTAATCCCAGC***ACTTTGGGAG DCAF10 3305
TGGGCATGGT***GGCTCATGCCTGTAATCCCAGC***ACTTTGGGAG DCAF10 4559
CAGGCACGGT***GGCTCATGCCTGTAATCCCAGC***TACGCAGGAG DCLRE1C 2966
CAGGCACGGT***GGCTCATGCCTGTAATCCCAGC***ACTTTGGGAG DDOST 1782
GGGGTGTGGT***GGCTCATGCCTGTAATCCCAGC***ACTTTGGGAG DHODH 1709
TGGGCGTGGT***GGCTCATGCCTGTAATCCCAGC***ACTTTGGGAG DNAJC22 1554
TGGGCACTGT***GGCTCATGCCTGTAATCCCAGC***ACTTTGGGAG DNAL1 4925
CAGGCGTGAT***GGCTCATGCCTGTAATCCCAGC***TACTCGGGAG DSCR6 1706
TGGGTGCAGT***GGCTCATGCCTGTAATCCCAGC***CAGCACTTTG ERBB3 5104
CAGGGGCGGT***GGCTCATGCCTGTAATCCCAGC***ACTTTGGGAG FADS6 1777
CGGGTATGGT***GGCTCATGCCTGTAATCCCAGC***ATTTTGGGAG FAM161A 2785
CGGGTGCAGT***GGCTCATGCCTGTAATCCCAGC***ACTTTGGGAG FAM227A 4981
CGGGCACAGT***GGCTCATGCCTGTAATCCCAGC***ACTTTGGGAG FAM84B 3626
CGGATGTGGT**GGCTCATGCCTGTAATCCCAGC**ACTTTGGGAG FBLIM1 2126
CAGGTGCGGT***GGCTCATGCCTGTAATCCCAGC***ACTTTAGGAG FBLX22 1411
CAGGCACAGT***GGCTCATGCCTGTAATCCCAGC***ACTTTGGGAG FBXO27 1535
CGGGCATAGT***GGCTCATGCCTGTAATCCCAGC***CCTTGGGAGG FGD4 7619
CAGGCATGGT***GGCTCATGCCTGTAATCCCAGC***ACTTTGGGAG FKBP14 1515
TGGGTGTGGT***GGCTCATGCCTGTAATCCCAGC***ACTTTGGGGG FKBP14 2129
CAGGTGTGGT***GGCTCATGCCTGTAATCCCAGC***ACTTTGGGAG FKBP5 7114
CTGGCACAGT***GGCTCATGCCTGTAATCCCAGC***ACTTTGGGAG FXN 3288
TGAGTGTGGT***GGCTCATGCCTGTAATCCCAGC***ACTTTGGGAG GDPD1 1559
CCGGCGCGGT***GGCTCATGCCTGTAATCCCAGC***ACTTTGGGAG GEMIN8 2172
TGGGTGCGGT***GGCTCATGCCTGTAATCCCAGC***ACTTTGGGAG GGT6 1956
TGTGTGCAGT***GGCTCATGCCTGTAATCCCAGC***ACTTTGGCAG GK5 3808
CAAGTACAGT***GGCTCATGCCTGTAATCCCAGC***ACTTTGGGAG GK5 6355
CGGGCATGGT***GGCTCATGCCTGTAATCCCAGC***ACTTTGGGAG GLB1L 2224
CGGGCGCGGT***GGCTCATGCCTGTAATCCCAGC***ACTTTGGGAG GOLGA3 7240
TGGGCGCAGT***GGCTCATGCCTGTAATCCCAGC***ATTTTGGGAG GP2 1877
CGGGCGCGGT***GGCTCATGCCTGTAATCCCAGC***ACTTTGGGAG GPR65 3309
TGGGTGCGGT***GGCTCATGCCTGTAATCCCAGC***ACTCTGGGAG GPR82 2664
CTGGTACAGT***GGCTCATGCCTGTAATCCCAGC***ACTTTGGGAG GPRIN2 6676
CAGGCATGGT***GGCTCATGCCTGTAATCCCAGC***ACTTTTCGGA GTPBP10 1873
GGCTAGGTGT***GGCTCATGCCTGTAATCCCAGC***ACTATGGGAG H6PD 5754
GCCAGGTGGT***GGCTCATGCCTGTAATCCCAGC***ACTTTGGGAG HM13 1745
CGGGCGCAGT***GGCTCATGCCTGTAATCCCAGC***ACTTTGGGAG IFIT3 1864
CAGGCGCAGT***GGCTCATGCCTGTAATCCCAGC***ACTTTGGGAG IYD 1658
TGAGCATGGT***GGCTCATGCCTGTAATCCCAGC***ACTTTGGGAG KIAA1456 2536
CGGGCGCGGT***GGCTCATGCCTGTAATCCCAGC***ACTTTGGGAG KIF11 3598
TGGGCATGGT***GGCTCATGCCTGTAATCCCAGC***ACTTTGGGAG KLHL23 2570
CGGATGTGGT***GGCTCATGCCTGTAATCCCAGC***AGTTCAGGAT KPNA1 5711
TGGGCGCGAT***GGCTCATGCCTGTAATCCCAGC***ACTGTGGGAG KREMEN1 2199
CGGGCGCGGT***GGCTCATGCCTGTAATCCCAGC***ACTTTGGGAG KREMEN1 2792
CAGGTGCAGT***GGCTCATGCCTGTAATCCCAGC***ATTTTGGTAG LAX1 2057
CGGGCACGGT***GGCTCATGCCTGTAATCCCAGC***ACTTTGGGAG LILRA6 2201
CAGGCACAGT***GGCTCATGCCTGTAATCCCAGG***CGCCGTGGCT LIMD1 5735
CGGGCGTGGT***GGCTCATGCCTGTAATCCCAGC***ACTTTGGGAG LIMS1 3931
CAGGTGCGGT***GGCTCATGCCTGTAATCCCAGC***ACTATGGGAG LMOD3 3224
CCGGTGCGGT***GGCTCATGCCTGTAATCCCAGC***ACTGTAGGCG LMOD3 3993
TGGGTGCGGT***GGCTCATGCCTGTAATCCCAGC***ACTTCGGGAG METTL6 1188
CGGGCACGGT***GGCTCATGCCTGTAATCCCAGC***ATTTTGGGAG MR1 3664
TGGGCGTGGT***GGCTCATGCCTGTAATCCCAGC***ACTTTGGGAG MREG 1540
TGGGTGTGGT***GGCTCATGCCTGTAATCCCAGC***ACTTTGGGAG MRPS25 1609
