# Supplementary material for: The Binding Sites of miR-619-5p in the mRNAs of Human and Orthologous Genes
Source: BMC Genomics. 2017 Jun 1;18:428. doi: 10.1186/s12864-017-3811-6 (PMC5452331; doi:10.1186/s12864-017-3811-6)
Supplement: Supplementary file 3 — Variation of nucleotide sequences of mRNA region with miR-619-5p binding sites of genes from IFIT3 to SLC26A4 (Conservative binding sites are in bold) (PDF 139 kb) [file 12864_2017_3811_MOESM3_ESM.pdf]

Figure 3 Variation of nucleotide sequences of mRNA region with miR-619-5p binding sites of genes from *IFIT3* to *SLC26A4* (Conservative binding sites are in bold)

CAGGCATGGT***GGCTCATGCCTGTAATCCCAGC***ACTTTGGGAG MSH3 4139
CGGGTGTGGT***GGCTCATGCCTGTAATCCCAGC***ACTTTGGGAG NANOS1 3219
CAGGTGTCGT***GGCTCATGCCTGTAATCCCAGC***ACTTTGGGAG NCMAP 2259
TGGGCGTGGT***GGCTCATGCCTGTAATCCCAGC***ACTTTGGGAG NDUFAF7 1697
CCGATGCAGT***GGCTCATGCCTGTAATCCCAGC***ACTTTGAGAG NDUFC2 1646
CGGGCGCAGT***GGCTCATGCCTGTAATCCCAGC***ACTTTGGGAG NLN 4215
CAGGCGCAAT***GGCTCATGCCTGTAATCCCAGC***ACTTTGGGAG NRIP2 2075
AGGCGCGGTG***GGCTCATGCCTGTAATCCCAGC***ACTTTGGGAG NSL1 3063
CGGGCATAGT***GGCTCATGCCTGTAATCCCAGC***ACTTTGGGAG NXPE3 7447
CGGGCACAGT***GGCTCATGCCTGTAATCCCAGC***ACTTTGGGAG OPTN 2332
CGGGTGCAGT***GGCTCATGCCTGTAATCCCAGC***ACTTTGGGAG PAG1 8156
TGGATGCAGT***GGCTCATGCCTGTAATCCCAGC***ACTTTGGGAG PAQR5 4439
CGGGCGCGGT***GGCTCATGCCTGTAATCCCAGC***ACTTTGGGAG PARK2 3729
TGGGCGTGGC***GGCTCATGCCTGTAATCCCAGC***ACTTTGGCAG PBLD 2077
CGGGCACAGT***GGCTCATGCCTGTAATCCCAGC***ACTTTGGGAG PCGF5 5089
CAGGACTGCT***GGCTCATGCCTGTAATCCCAGC***ACTCTGGGAG PCSK5 8613
CGGGCATGGT***GGCTCATGCCTGTAATCCCAGC***ACTTTGGGAG PDAP1 1926
TGGGCACGGT***GGCTCATGCCTGTAATCCCAGC***ACTTTGGGAG PDCD4 3221
TGAGCACAAT***GGCTCATGCCTGTAATCCCAGG***CTCCCTACTT PEX2 3056
CAGGCGCAGT***GGCTCATGCCTGTAATCCCAGC***ACTTTGGAAG PGPEP1 1476
CAGGCACGGT***GGCTCATGCCTGTAATCCCAGC***ACTTTGGGAG PIK3R2 3345
CAGGCATGGT***GGCTCATGCCTGTAATCCCAGC***ACTTTGGGAG PNPLA1 1991
TGGGTGAGGT***GGCTCATGCCTGTAATCCCAGC***ACTTTGGGGA PODNL1 1876
CAAGTGTGGT***GGCTCATGCCTGTAATCCCAGC***ACTTTGCGAG POFUT1 4679
CGGGTGCAGT***GGCTCATGCCTGTAATCCCAGC***ACTTTGGGAG POLH 5550
CAGGCACAGT***GGCTCATGCCTGTAATCCCAGC***ACTTTGAGAG PPM1K 2192
CAGGTGCAGT***GGCTCATGCCTGTAATCCCAGC***ACTTCAGGAG PPP1R12B 5156
CGGGCATGGT***GGCTCATGCCTGTAATCCCAGC***ACTTTGGGAG PRRG4 998
CGGGTGCGGT***GGCTCATGCCTGTAATCCCAGC***ACTTTGGCCG PSMB2 2925
TGAGTGCAGT***GGCTCATGCCTGTAATCCCAGC***ACTTAGGGTG PTCD3 4116
GGGGCACGGT***GGCTCATGCCTGTAATCCCAGC***ACTTTGGGAG PTK6 2233
CAGGCACGGT***GGCTCATGCCTGTAATCCCAGC***ACTTTGGGAG QRFPR 1949
CAGTCGTGGT***GGCTCATGCCTGTAATCCCAGC***ACTTTGCAAG RAB11FIP1 4928
TAGGCATTGT***GGCTCATGCCTGTAATCCCAGC***ACTTTGGGAG RAB3IP 3975
CAGGTGCGAT***GGCTCATGCCTGTAATCCCAGC***ACTTTGGGAG RAB3IP 7022
CGGGCATGGT***GGCTCATGCCTGTAATCCCAGC***ACTTGGGGAG RAB7L1 1693
TGGGTGTGGT***GGCTCATGCCTGTAATCCCAGC***ACTGTGGGAG RBBP9 1818
CGGGCGCAGT***GGCTCATGCCTGTAATCCCAGC***ACTTTGAGAG SCN11A 5871
CAGGTGTGGT***GGCTCATGCCTGTAATCCCAGC***ACTTTGAGAG SEPT11 4033
GGGGTGTGGT***GGCTCATGCCTGTAATCCCAGC***ACTTTGGGAG SEPT14 1575
CGGGCGCTGT***GGCTCATGCCTGTAATCCCAGC***ACTTTGGGAG SGTB 3142
TGGGTGCCGT***GGCTCATGCCTGTAATCCCAGC***ACTTTGGAGG SH3GLB1 4856
CAGGCACAGT***GGCTCATGCCTGTAATCCCAGC***ACTTTGGGAG SLC15A2 4333
CGGGCACGGT***GGCTCATGCCTGTAATCCCAGC***ACTTTGGGGG SLC17A5 2389
CAGGTGCAGT***GGCTCATGCCTGTAATCCCAGC***ACGTTGGGAG SLC26A2 5066
CAGGCGCGGT***GGCTCATGCCTGTAATCCCAGC***ACTTTGGGAG SLC26A4 4210
CGGGCTCAGT***GGCTCATGCCTGTAATCCCAGC***ACTTTGGGAG SLC28A2 2196
CCGGCACGGT***GGCTCATGCCTGTAATCCCAGC***ACTTTGGGAG SLC7A11 6304
CGGGCGCAGT***GGCTCATGCCTGTAATCCCAGC***ACTTTGGGAG SLC7A14 8487
CAGGCGCGGT***GGCTCATGCCTGTAATCCCAGC***ACTTTGGGAG SNX22 902
CAGGCATGGT***GGCTCATGCCTGTAATCCCAGC***ACTTTGGGAG SOWAHC 3417
TGGGTGCTGT***GGCTCATGCCTGTAATCCCAGC***ACTTTGGGAG SPATA13 5020
TGGGCGCGGT***GGCTCATGCCTGTAATCCCAGC***ACTTTGGGAG SPATA5 5648
