# Supplementary material for: The Binding Sites of miR-619-5p in the mRNAs of Human and Orthologous Genes
Source: BMC Genomics. 2017 Jun 1;18:428. doi: 10.1186/s12864-017-3811-6 (PMC5452331; doi:10.1186/s12864-017-3811-6)
Supplement: Supplementary file 4 — Variation of nucleotide sequences of mRNA region with miR-619-5p binding sites of genes from LC28A2 to ZNF841 (Conservative binding sites are in bold). The data given in the Additional files 1, 2, 3 and 4 demonstrate the variability of the nucleotides before and after the binding sites of miR-619-5p, which is shown in the Weblogo schemes in the table 8. (PDF 151 kb) [file 12864_2017_3811_MOESM4_ESM.pdf]

Figure 4 Variation of nucleotide sequences of mRNA region with miR-619-5p binding sites of genes from *LC28A2* to *ZNF841* (Conservative binding sites are in bold)

TGGGCGTTGT***GGCTCATGCCTGTAATCCCAGC***ACTTTGGGAG SPATS2 3332
CCAGCGCAGT***GGCTCATGCCTGTAATCCCAGC***ACTTTGGGAG SPN 5287
CAGGTGTGGT***GGCTCATGCCTGTAATCCCAGC***ACTTTGGGAG STAC2 2241
TGGGAGAGGT***GGCTCATGCCTGTAAATCCCAG***CACTTTGGTA SYNJ2BP 1298
TGGGCATGGT***GGCTCATGCCTGTAATCCCAGC***ACTCTAGGAG SYNJ2BP 4175
TGAGCCCGGT***GGCTCATGCCTGTAATCCCAGC***ACTTTCAGAG TCEB1 1964
TGGGCATGGT***GGCTCATGCCTGTAATCCCAGC***ACATTGGGAA TIGD6 3439
CAGGCGCAGT***GGCTCATGCCTGTAATCCCAGC***ACTTTGGGAT TMEM156 1593
CGGGCGCACT***GGCTCATGCCTGTAATCCCAGC***ATTTTGGAAG TMEM19 3510
CAGGTATGGT***GGCTCATGCCTGTAATCCCAGC***ACTTTGGGAG TMEM213 875
TGGGCATGGT***GGCTCATGCCTGTAATCCCAGC***ACTTTGAGAG TMEM213 1190
TGGGCACGGT***GGCTCATGCCTGTAATCCCAGC***ACTTTGGGAG TMEM50B 1026
CGGGCACGGT***GGCTCATGCCTGTAATCCCAGC***ACTTTGGGAG TMEM56 1243
CAGGTGCTGT***GGCTCATGCCTGTAATCCCAGC***ACTTTGGGAG TMF1 4736
TGGGCCCAGT***GGCTCATGCCTGTAATCCCAGC***ACTTTGGGAG TMOD2 7816
CAGGTGTAGT***GGCTCATGCCTGTAATCCCAGC***ACTTTGGGAG TNFRSF10A 1621
CACATGCGGT***GGCTCATGCCTGTAATCCCAGC***ACTTTGATAG TNFRSF10D 1532
CGGGTGCAGT***GGCTCATGCCTGTAATCCCAGC***ACTTTGGGAG TOP3A 3814
CCAGCATGGT***GGCTCATGCCTGTAATCCCAGC***ATTTTGGGAG TPRG1L 1754
CTGGCTTGGT***GGCTCATGCCTGTAATCCCAGC***ACTTTGGGAG TRIM72 1885
GGGGCATGGT***GGCTCATGCCTGTAATCCCAGC***ACTTCGGGAG TRPM7 8079
CAGGTGCAGT***GGCTCATGCCTGTAATCCCAGC***ACTTTGGGAG TRPM7 8221
TGGGCGCAGT***GGCTCATGCCTGTAATCCCAGC***ACTTTGGGAG TXNDC15 2460
CAGGTGCCAT***GGCTCATGCCTGTAATCCCAGC***ACTTTGGGAG TYW5 3692
CGGGGGTGGT***GGCTCATGCCTGTAATCCCAGC***ACTTTGGGAG UACA 6120
CGGGCGCGGT***GGCTCATGCCTGTAATCCCAGC***ACTTTGGGAG UBIAD1 2881
CGGGTGCAAT***GGCTCATGCCTGTAATCCCAGC***ACTTTGGGAG UBXN2A 1665
TGGGCGTGGG***GGCTCATGCCTGTAATCCCAGC***ACTTTGGGAG UPK1B 1513
AAGGCATGGT***GGCTCATGCCTGTAATCCCAGC***ACTTTGGGAG UQCRB 1269
CGAGCACAGT***GGCTCATGCCTGTAATCCCAGC***ATTTTGGGAG VHL 3764
CGGCCGCGGC***GGCTCATGCCTGTAATCCCAGC***ACTTTGGGAG VHL 3898
CAGAGACTGT***GGCTCATGCCTGTAATCCCAGC***ACTTTGGAGG VWA2 3366
TGGACATGGT***GGCTCATGCCTGTAATCCCAGC***ACTTTCAGAG WDR73 1736
TGGGCGCGGT***GGCTCATGCCTGTAATCCCAGC***ACTTTGGGAG XIAP 5681
TGGGCGTGGT***GGCTCATGCCTGTAATCCCAGC***TACTTGGGAG XIAP 5815
TGGGTATGGT***GGCTCATGCCTGTAATCCCAGC***ACTTTGGGAG YAE1D1 1548
CGGGCGTGGA***GGCTCATGCCTGTAATCCCAGC***ACTTTGGGAG ZBTB24 4842
CCGGCATGGT***GGCTCATGCCTGTAATCCCAGC***ACTTTGGAAG ZC3H12D 2812
TGGGTGTGGT***GGCTCATGCCTGTAATCCCAGC***ACTTTGGGAG ZDHHC20 3390
TGGGCCCGGT***GGCTCATGCCTGTAATCCCAGC***ACTTTGGGAG ZFP30 3463
CGGCCGCGGT***GGCTCATGCCTGTAATCCCAGC***ACTTTGGGAG ZNF114 1827
CGGGCTTGGT***GGCTCATGCCTGTAATCCCAGC***ACTTTGAGAG ZNF197 3446
CAGGTGCTCT***GGCTCATGCCTGTAATCCCAGC***ACTTTGGGAG ZNF320 5534
TGGGTGTGGT***GGCTCATGCCTGTAATCCCAGC***ACTTTGGGAG ZNF429 2081
CTGGCACGAT***GGCTCATGCCTGTAATCCCAGC***ACTTTGGGAG ZNF445 8820
CAGGCTTGGT***GGCTCATGCCTGTAATCCCAGC***ACTTTGGGAG ZNF461 3087
TGGGTGCGGT***GGCTCATGCCTGTAATCCCAGC***ACTTTGGGAG ZNF549 3736
CAGGCGCCAT***GGCTCATGCCTGTAATCCCAGC***ACTTTGGGAG ZNF557 4791
TGGACGCAGT***GGCTCATGCCTGTAATCCCAGC***ACTTTGGTAG ZNF626 4620
TGAGCCCGGT***GGCTCATGCCTGTAATCCCAGC***ACTTTGGGAG ZNF667 3240
TAGATGTGGT***GGCTCATGCCTGTAATCCCAGC***ACTTTGGGAG ZNF716 2799
CGGGAGCAGT***GGCTCATGCCTGTAATCCCAGC***ACTTTGGGAG ZNF780B 5415
TGGGCACAGT***GGCTCATGCCTGTAATCCCAGC***ACTTTGGGAG ZNF84 4920
CATGAGGTCT***GGCTCATGCCTGTAATCCCAGC***ACTTTGGGAG ZNF841 3422
